# Modeling the Relationship of Groundwater Salinity to Neonatal and Infant Mortality From the Bangladesh Demographic Health Survey 2000 to 2014

**DOI:** 10.1029/2019GH000229

**Published:** 2020-02-17

**Authors:** Abu Mohd Naser, Qiao Wang, Mohammad Shamsudduha, Gnanaraj Chellaraj, George Joseph

**Affiliations:** ^1^ Emory Global Diabetes Research Center, Hubert Department of Global Health, Rollins School of Public Health Emory University Atlanta GA USA; ^2^ World Bank Washington DC USA; ^3^ Institute for Risk and Disaster Reduction University College London London UK; ^4^ Department of Geography University of Sussex Brighton UK

**Keywords:** Drinking water salinity, electrical conductivity, infant mortality, neonatal mortality, water calcium, water sodium

## Abstract

We evaluated the relationship of drinking water salinity to neonatal and infant mortality using Bangladesh Demographic Health Surveys of 2000, 2004, 2007, 2011, and 2014. Point data of groundwater electrical conductivity (EC)— a measure of salinity—were collated from the Bangladesh Water Development Board and digitizing salinity contour map. Data for groundwater dissolved elements (sodium, calcium, magnesium, and potassium) data came from a national hydrochemistry survey in Bangladesh. Point EC and dissolved minerals data were then interpolated over entire Bangladesh and extracted to each cluster location, the primary sampling unit of Bangladesh Demographic Health Surveys. We used restricted cubic splines and survey design‐specific logistic regression models to determine the relationship of water salinity to neonatal and infant mortality. A U‐shaped association between drinking water salinity and neonatal and infant mortality was found, suggesting higher mortality when salinity was very low and high. Compared to mildly saline (EC ≥0.7 and < 2 mS/cm) water drinkers, freshwater (EC < 0.7 mS/cm) drinkers had 1.37 (95% CI: 1.01, 1.84) times higher neonatal mortality and 1.43 (95% CI: 1.08, 1.89) times higher infant mortality. Compared to mildly saline water drinkers, severe‐saline (EC ≥10 mS/cm) water drinkers had 1.77 (95% CI: 1.17, 2.68) times higher neonatal mortality and 1.93 (95% CI: 1.35, 2.76) times higher infant mortality. We found that mild‐salinity water had a high concentration of calcium and magnesium, whereas severe‐salinity water had a high concentration of sodium. Freshwater had the least concentrations of salubrious calcium and magnesium.

## Key Points

- Water salinity indicates all dissolved ions in water: The common cations are sodium, potassium, calcium, and magnesium
- We found a U‐shaped association between drinking water salinity and neonatal and infant mortality, suggesting higher mortality when drinking water salinity was very low and high
- We found mild‐salinity water had a high concentration of calcium and magnesium, whereas severe‐salinity water had a high level of sodium

## 1 Introduction

Globally, groundwater is widely used for drinking, food preparations, irrigation, and industrial purposes (Van Weert et al., 2009). Intensive groundwater extraction for irrigation and municipal water supplies in many coastal areas around the world has resulted in lower groundwater quality by inducing saltwater intrusion (Ferguson & Gleeson, 2012). Rising sea levels due to global climate change are contributing to increasing groundwater salinity in many coastal areas (Van Weert et al., 2009).

Water salinity is measured as electrical conductivity (EC), which indicates the ability of water to conduct an electrical current where the dissolved elements are the conductors (J. Rhoades, 1996). During the slow passage through the rocks and soil, elements dissolve in groundwater (National Ground Water Association, 2017). The concentrations of dissolved elements in saline water varies significantly from one location to another, both in terms of specific elements and concentration levels (Van Weert et al., 2009). Bangladesh is the largest delta in the world and was formed by the active sediment deposits from the Himalayas carried by the Ganges, Brahmaputra, and Meghna rivers (France‐Lanord et al., 2013). Shallow groundwater in the southeast and central Bangladesh has elevated arsenic; however, saline groundwater in southwest coastal Bangladesh has been linked to health problems such as high blood pressure (Talukder et al., 2016). Saltwater intrusion, a process of groundwater salinity due to movement of fresh‐saline groundwater interface inland along the shores (Cooper, 1964), has increased groundwater salinity in coastal Bangladesh (Shamsudduha et al., 2019).

A national groundwater quality survey 2009 conducted by UNICEF indicates the major elements (median concentration > 1 mg/L) in groundwater are calcium, sodium, chlorine, magnesium, and potassium (UNICEF Bangladesh & Bangladesh Bureau of Statistics, 2009). The majority (97%) of the rural population in Bangladesh depend on groundwater for drinking and domestic water use (British Geological Survey, 2001). Elements present in groundwater in Bangladesh may be an important source of maternal mineral intake, which can influence neonatal and infant death (Kovacs, 2016; Prentice, 2003). Increased concentrations of sodium in saline water, a detrimental mineral if consumed in large amounts, have been associated with a higher prevalence of gestational hypertension among pregnant women in coastal Bangladesh (Khan et al., 2014). Neonatal and infant deaths have remained high in Bangladesh, although it has declined in recent years (Ahsan et al., 2015). However, limited data exist on the role of drinking water salinity or element contents on neonatal and infant death. In this study, we evaluated the relationship between groundwater salinity with neonatal and infant deaths in Bangladesh.

## 2 Materials and Methods

### 2.1 Health Outcomes and Covariate Data Sources

We compiled data from the Bangladesh Demographic Health Survey (BDHS) for the year of 2000, 2004, 2007, 2011, and 2014. In each BDHS, households were recruited from rural and urban areas representing the seven administrative divisions. The United States Agency (USAID) funded the BDHS data collections, as part of an effort to collect population health, nutrition, mortality, and fertility indicators every 4 years in selected low‐ and middle‐income countries (The DHS Program, n.d.), and the National Institute of Population Research and Training of the government of Bangladesh (GOB) implemented the surveys. Clusters represented the primary sampling unit for each BDHS year from which representative households were selected for interviews.

BDHS surveys interviewed women of 12–49 years and asked for live birth, child death, sex of the child, birth order, age, and marital status of the mother during the time of delivery. Neonatal death was defined if a child died within the first month of life, and infant death was defined if the child died before the first birthday. The primary outcomes for our analyses were neonatal and infant mortality that occurred within the 3‐year window before the interview year. Other information such as parent education, the rural or urban residence, asset information, sanitation, and households primary drinking water sources were also collected. Household wealth quintiles were calculated from the household assets by principal component analysis and were available in each year's BDHS data set.

### 2.2 Groundwater EC Data

**Figure 1:**
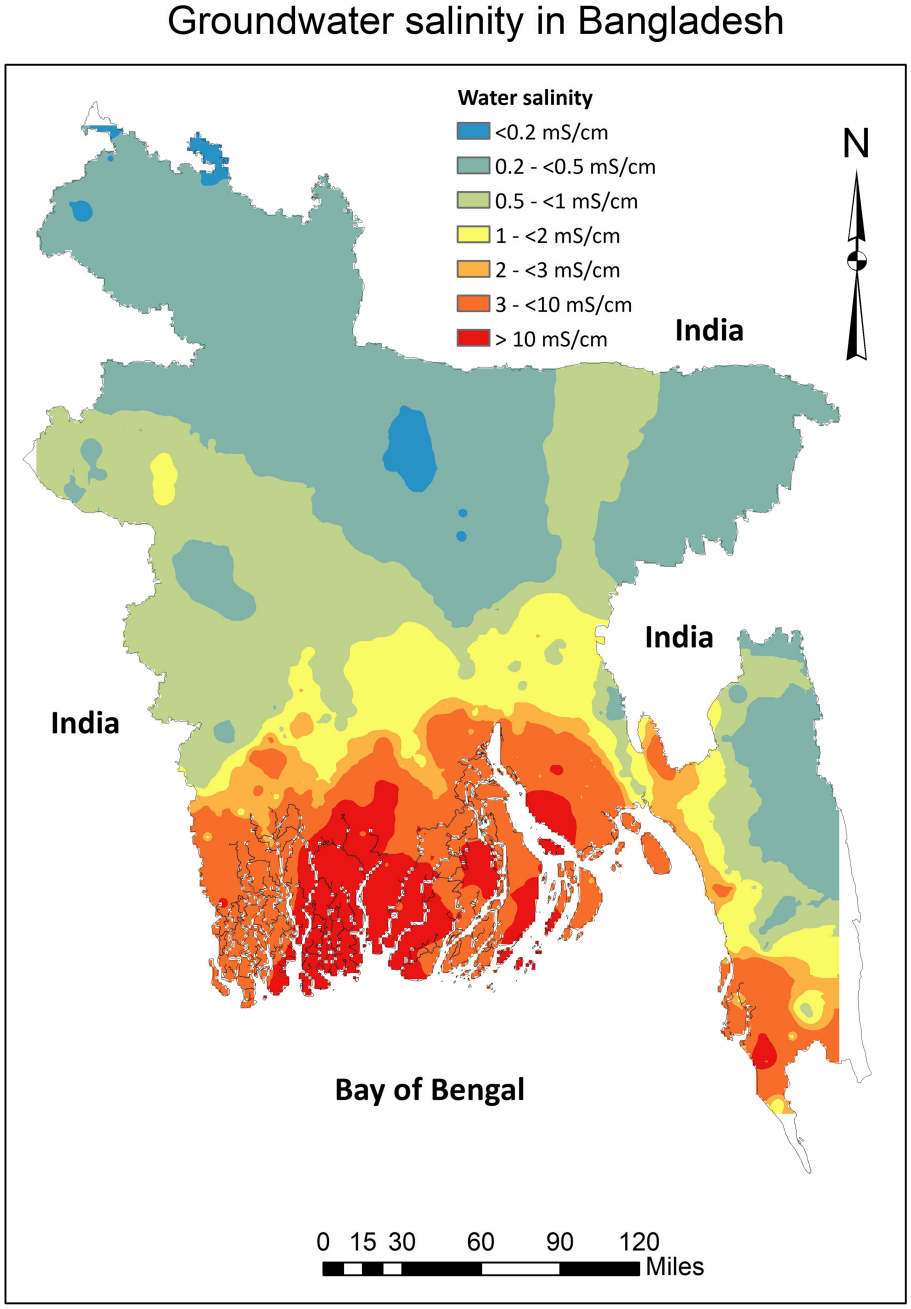
Drinking water salinity (electrical conductivity) levels across Bangladesh.

Groundwater EC data were collected from two sources. First, we collated groundwater EC data from a monitoring network of 461 boreholes established between 2011 and 2013 under a regional‐scale hydrogeology study conducted in 19 coastal districts by the Bangladesh Water Development Board (Zahid et al., 2013; Zahid et al., 2016). Second, we digitized and georeferenced the contoured map of groundwater EC (A. A. Rahman & Ravenscroft, 2003) for shallow aquifers and extracted EC point data at 102 locations predominantly in the northern part of Bangladesh where EC measurement is limited. We then interpolated the point EC data (*n* = 563) over entire Bangladesh using the inverse distance weighting (IDW) algorithm in the ArcGIS environment (Shamsudduha et al., 2019). We then extracted interpolated EC values for each cluster location to get groundwater salinity data for each of the BDHS clusters (Figure 1).

### 2.3 Groundwater Dissolved Elements and Arsenic

The groundwater arsenic and dissolved mineral concentrations data are from the British Geological Survey (BGS). In collaboration with the Department of Public Health Engineering (DPHE) of the GOB, BGS conducted a groundwater chemicals survey in 3,534 wells representative of entire Bangladesh during the 1998–1999 period (Kinniburgh & Smedley, 2001). Arsenic was measured using hydride generation‐atomic fluorescence spectrometry, and groundwater sodium, potassium, calcium, and magnesium were measured by inductively coupled plasma‐atomic emission spectrometry (Kinniburgh & Smedley, 2001). We interpolated the groundwater arsenic, sodium, potassium, calcium, and magnesium over entire Bangladesh using the BGS‐DPHE data using the IDW in ArcGIS. Concentrations of these elements for BDHS clusters were extracted for all years using the cluster geocodes and were considered as mineral exposure in drinking water in our analyses.

### 2.4 Statistical Analyses

After pooling the five BDHS data sets, we calculated revised sample weights following denormalization of the standard weights (ICF International, 2012). We calculated the survey‐weighted proportions of neonatal and infinity mortality for each 100 live births and compared these proportions among different categories of exposure and covariates with respect to reference categories using the chi‐square test.

Since our key objective was to evaluate the association between groundwater salinity and neonatal or infant deaths, we restricted all statistical modeling among the subpopulation of participants who reported tubewells as their primary source of drinking water in all BDHS surveys. The nontubewell water drinkers reported they consumed water mainly from the piped water sources. However, further details of the source of piped water supply—whether from groundwater or surface—water were absent. We modeled groundwater salinity as continuous variable, categorical variable, and restricted cubic splines. The distribution of groundwater EC is right skewed (Figure S1 in the supporting information). Since the EC data contained outliers, we winsorized the data at 95th percentile distribution when modeled EC as continuous exposure (Tan & Tabatabai, 1988).

#### 2.4.1 Association Between Groundwater Salinity and Mortality

To visually assess the relationship between drinking water salinity with neonatal and infant mortality, we plotted restricted cubic spline plots (Orsini & Greenland, 2011) to illustrate how the odds ratios of neonatal and infant mortality changes with the increasing level of drinking water EC. We used four knots at EC cut‐points of 5th, 35th, 65th, and 95th percentiles to create the restricted cubic spline plots (Harrell, 2015). Restricted cubic plots assume cubic polynomials in segments after the first knot and before the last knot (Croxford, 2016). Hence, the spline plots can identify nonlinear association between 5th and 95th percentiles distribution of the EC data. We used EC value of 0.5 mS/cm as the reference against which odds ratios for restricted cubic plots were calculated.

We then determined the associations of drinking water salinity categories with odds of neonatal and infant mortality using survey design‐specific logistic regression models. There are no official guidelines for drinking water in Bangladesh considering the human health perspective. We used the water salinity categories defined by the Food and Agricultural Organization of the United Nations for irrigation water: freshwater (EC < 0.7 mS/cm), mild‐salinity (EC ≥ 0.7 and < 2 mS/cm), moderate‐salinity (EC ≥ 2 and < 10 mS/cm), and severe‐salinity (EC ≥ 10 mS/cm; Rhoades et al., 1992). Odds ratios were determined with respect to mild‐saline water (reference group).

For all models described above, we sequentially reported odds ratios of unadjusted models (Model 1) and models partially adjusted for maternal age, birth order, and sex of the child (Model 2). We then additionally adjusted for both parents’ years of education, rural or urban residence, household wealth score, improved sanitation, child's birth year, maternal marital status, depth of tubewells, arsenic concentrations in tubewell water, geographical division, and river basin for the BDHS cluster (Model 3). We adjusted for arsenic concentrations because Bangladesh National Drinking Quality Survey 2009 demonstrated high groundwater arsenic coexist with some high salinity areas in southwest coastal Bangladesh (UNICEF Bangladesh & Bangladesh Bureau of Statistics, 2009), and groundwater arsenic was associated with higher infant mortality in Bangladesh (A. Rahman et al., 2010). The purpose of adjustment by the “geographical division” and “river basin” was to account for spatial factors, including the hydrogeological parameters, socioeconomical conditions, and health care utilization of the population that may influence water salinity and mortality. We used the birth order of the child, maternal age, depth of tubewell, wealth score, and parent's year of education as continuous variables. However, sex of the child, marital status of the mother, rural or urban residence, improved sanitation, child's birth year, geographical division, and river basin were used as categorical variables.

#### 2.4.2 Association Between Groundwater Dissolved Elements and Mortality

We first determined the association between groundwater EC and each of the interpolated elements (sodium, calcium, magnesium, and potassium) in water using the restricted cubic splines using four knots described previously. We then determined the association between each of the groundwater elements with neonatal and infant mortality using the restricted cubic splines by implementing four knots described previously at cut‐points of 5th, 35th, 65th, and 95th percentiles data for each element. We used the standard values (200 mg/L for sodium; 75 mg/L for calcium; 35 mg/L for magnesium, and 12 mg/L for potassium) of the Department of Environment of the GOB (Department of Environment, 1997) as the reference values against which odds ratios for restricted cubic plots for each water dissolved element were calculated.

## 3 Results

### 3.1 Number of Neonatal and Infant Deaths

A total of 185,466 live births, 12,053 neonatal, and 16,015 infant deaths was reported in five BDHS. We calculated 995 neonatal and 1,183 infant deaths within the 3 years of five BDHS surveys (Table 1). Households with 862 neonatal and 1,022 infant deaths reported tubewells as the primary source of drinking water (Table 1).

**Table 1:** Number of Neonatal and Infant Death Analyzed From Different Bangladesh Demographic Health Surveys

Number of deaths	DHS year					
	2000	2004	2007	2011	2014	Combined
Number of live births	31,925	33,475	30,527	45,844	43,695	185,466
Number of deaths						
Neonatal	2,549	2,421	2,005	2,688	2,390	12,053
Infant	3,529	3,288	2,664	3,481	3,053	16,015
Number of live births within 3 years of DHS year	4,114	4,233	3,751	5,191	4,899	22,188
Number of deaths within 3 years of DHS year						
Neonatal	215	211	168	213	188	995
Infant	253	267	203	247	213	1,183
Number of child deaths within 3 years of DHS year whose mother reported consuming tubewell water						
Neonatal	190	170	153	190	159	862
Infant	224	214	182	222	180	1,022

### 3.2 Distribution of Neonatal and Infant Death Across Covariate

We found that neonatal and infant mortality decreased over the period of successive DHS years (Table 2). Of the participants, 60% consumed freshwater, 20% consumed mild‐salinity water, 17% consumed moderate‐saline water, and 3% consumed severe‐saline water. We found 657 neonatal and 771 infant deaths among freshwater drinkers, 125 neonatal and 143 infant deaths among mild‐saline water drinkers, 153 neonatal and 194 infant deaths among moderate‐saline water drinkers, and 60 neonatal and 75 infant deaths among severe‐saline water drinkers. Male children and first‐borne children had higher neonatal and infant mortality. Children of <18 years of age mothers, unmarried mothers, whose parents had no institutional education, with unimproved household sanitation, rural residence, very poor households, particularly from Sylhet division, and those clusters were located in Brahmaputra‐Jamuna river basin had higher neonatal and infant mortality (Table 2). No differences in neonatal and infant mortality were observed across tubewell and nontubewell water drinkers and for different depths of tubewells.

**Table 2:** Distribution of Neonatal and Infant Mortalities Across Exposure Variables and Covariates

Variables	Odds (%) of neonatal death (95% CI)	*P* value^a^	Odds (%) of infant death (95% CI)	*P* value^a^
Death in total five BDHS year	4.5 (4.2, 4.9)	—	5.4 (5.0, 5.7)	—
Death in each BDHS year				
2000 (19%)	5.2 (4.5, 6.1)	ref	6.1 (5.4, 7.0)	ref
2004 (19%)	5.3 (4.5, 6.1)	0.616	6.6 (5.8, 7.6)	0.765
2007 (16%)	4.4 (3.6, 5.3)	0.124	5.4 (4.5, 6.3)	0.162
2011 (23%)	3.9 (3.3, 4.5)	0.010	4.5 (3.9, 5.2)	0.003
2014 (23%)	4.1 (3.5, 4.9)	0.001	4.7 (4.0, 5.4)	<0.001
Groundwater salinity categories				
Freshwater (60%)	5.1 (4.7, 5.6)	ref	6.0 (5.6, 6.6)	ref
Mid‐salinity water (20%)	3.5 (2.9, 4.3)	<0.001	4.0 (3.3, 4.8)	<0.001
Moderate‐salinity water (17%)	3.4 (2.8, 4.1)	<0.001	4.5 (3.8, 5.2)	<0.001
Severe‐salinity water (3%)	5.1 (3.8, 6.7)	0.984	6.5 (5.1, 8.1)	0.606
Sex of the child				
Male (51%)	4.8 (3.9, 4.8)	ref	5.6 (5.1, 6.1)	ref
Female (49%)	4.3 (3.9, 4.8)	0.008	5.2 (4.7, 5.7)	0.020
Maternal age at delivery				
<18 years (14%)	6.6 (5.6, 7.7)	ref	7.7 (6.6, 8.9)	ref
≥18 to 49 years (86%)	4.2 (3.9, 4.5)	<0.001	5.0 (4.7, 5.4)	<0.001
Birth order of the child				
First (35%)	5.6 (5.0, 6.2)	ref	6.3 (5.7, 7.0)	ref
Second (27%)	3.9 (3.3, 4.6)	<0.001	4.5 (3.8, 5.2)	<0.001
Third (17%)	3.8 (3.2, 4.6)	0.001	4.7 (3.9, 5.5)	0.004
Fourth or higher (21%)	4.3 (3.7, 5.0)	0.021	5.6 (5.0, 6.4)	0.716
Married mother				
No (1%)	6.4 (4.1, 9.8)	ref	6.8 (4.5, 10.3)	ref
Yes (99%)	4.5 (4.2, 4.8)	0.011	5.4 (5.0, 5.7)	0.016
Depth of tubewell				
<5 m (29%)	4.2 (3.7, 4.7)	ref	5.4 (4.8, 6.0)	ref
≥5 to <10 m (54%)	4.7 (4.2, 5.2)	0.144	5.4 (4.9, 5.9)	0.838
≥10 m (17%)	4.7 (3.9, 5.7)	0.451	5.4 (4.6, 6.4)	0.976
Well water arsenic				
<10	4.5 (4.0, 50)	ref	5.5 (4.9, 6.1)	ref
≥10 and < 50	5.0 (4.5, 5.7)	0.158	5.8 (5.2, 6.5)	0.479
≥50	4.2 (3.7, 4.8)	0.028	4.9 (4.3, 5.5)	0.009
Mothers education				
No institutional education (27%)	5.7 (5.1, 6.4)	ref	7.1 (6.3, 7.9)	ref
Primary‐level^b^ (30%)	4.4 (3.8, 5.0)	<0.001	5.2 (4.6, 5.9)	<0.001
Secondary‐level or higher (43%)	3.9 (3.5, 4.4)	<0.001	4.4 (4.0, 5.0)	<0.001
Fathers education				
No institutional education (34%)	5.5 (4.9, 6.2)	ref	6.7 (6.1, 7.5)	ref
Primary level^b^ (28%)	4.3 (3.8, 5.0)	0.003	5.2 (4.6, 5.8)	<0.001
Secondary level or higher (38%)	3.8 (3.3, 4.4)	<0.001	4.3 (3.8, 4.9)	<0.001
Tubewell as the primary drinking water source				
No (11%)	5.0 (4.1, 6.0)	ref	6.2 (5.3, 7.3)	ref
Yes (89%)	4.8 (4.1, 4.8)	0.168	5.3 (4.9, 5.7)	0.076
Improved household sanitation				
No (70%)	4.8 (4.4, 5.3)	ref	5.8 (5.4, 6.3)	ref
Yes (30%)	3.8 (3.3, 4.4)	<0.001	4.4 (3.8, 5.0)	<0.001
Residence				
Rural (78%)	4.7 (4.3, 5.1)	ref	5.5 (5.1, 5.9)	ref
Urban (22%)	4.0 (3.4, 4.6)	0.044	4.9 (4.3, 5.6)	0.070
Wealth quintile				
Very poor (31%)	5.2 (4.6, 5.9)	ref	6.4 (5.8, 7.1)	ref
Poor (16%)	5.0 (4.1, 5.9)	0.297	5.9 (5.0, 6.9)	0.262
Average (19%)	4.4 (3.6, 5.4)	0.049	5.0 (4.1, 6.0)	0.003
Less poor (23%)	4.0 (3.4, 4.7)	0.006	4.6 (3.9, 5.3)	<0.001
Least poor (11%)	3.4 (2.7, 4.3)	<0.001	4.1 (3.4, 5.0)	<0.001
Geographical division				
Barisal (6%)	3.9 (3.0, 5.0)	ref	4.9 (4.0, 6.1)	ref
Chittagong (22%)	3.4 (2.9, 4.0)	0.422	4.5 (3.9, 5.1)	0.264
Dhaka (32%)	4.9 (4.2, 5.6)	0.057	5.7 (5.0, 6.5)	0.194
Khulna (10%)	4.1 (3.3, 5.1)	0.903	4.5 (3.6, 5.5)	0.153
Rajshahi (18%)	5.1 (4.3, 6.0)	0.072	6.0 (5.1, 6.9)	0.256
Rangpur (5%)	3.5 (2.5, 4.8)	0.799	3.7 (2.7, 5.0)	0.343
Sylhet (8%)	6.7 (5.8, 7.7)	<0.001	7.9 (6.8, 9.0)	<0.001
River basin of BDHS cluster				
Brahmaputra‐Jamuna (48%)	4.8 (4.3, 5.3)	ref	5.8 (5.3, 6.3)	ref
Ganges_Padma (46%)	4.5 (4.0, 5.0)	0.014	5.1 (4.7, 5.7)	0.001
Meghna (6%)	3.0 (2.1, 4.3)	0.001	4.4 (3.4, 5.7)	0.006

a Chi‐square test

b Primary‐level education refers to ≤5‐year schooling

### 3.3 Drinking Water Salinity and Neonatal and Infant Mortality

**Figure 2:**
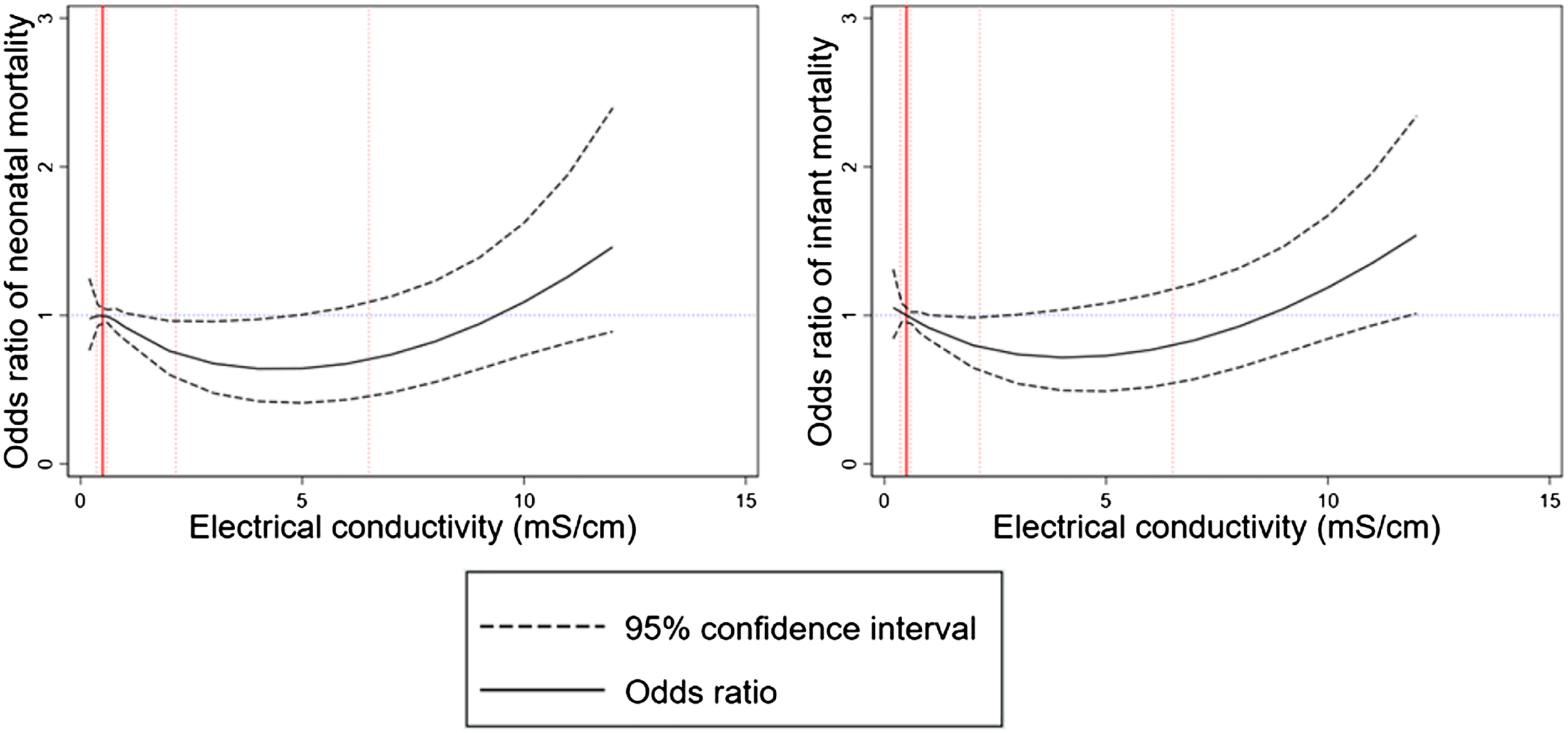
Restricted cubic spline plots (solid lines) and 95% confidence interval (dashed lines) for the association between drinking water electrical conductivity with neonatal and infant mortality. Restricted cubic splines were plotted at electrical conductivity cut‐points of 5th, 35th, 65th, and 95th percentiles. Restricted cubic spline plots were adjusted for maternal age, birth order, sex of the child, both parents’ years of education, rural or urban residence, household wealth score, improved sanitation, child's birth year, maternal marital status, depth of tubewells, arsenic concentrations in tubewell water, geographical division, and river basin. Distribution of electrical conductivity data at 10th, 50th (median), 75th, and 90th percentiles illustrated as red vertical dotted lines. Red solid vertical line (at electrical conductivity = 0.5 mS/cm) indicates the reference value against which odds ratios were calculated. Odds ratio = 1 denoted by blue dotted line.

The restricted cubic spline plots illustrate a U‐shaped nonlinear association between drinking water EC and neonatal and infant deaths, suggesting odds ratios for neonatal and infant deaths decreased initially and then increased again with the increasing levels of drinking water salinity as indicated by higher EC (Figure 2). Compared to the mild‐saline water drinkers, the risks for neonatal deaths were 1.37 (95% CI: 1.01, 1.84) times higher among the freshwater drinkers, 1.04 (95% CI: 0.75, 1.45) times among the moderate‐saline water drinkers, and 1.77 (95% CI: 1.17, 2.68) times higher among the severe‐saline water drinkers and the risks for infant deaths were 1.43 (95% CI: 1.08, 1.89) times higher among the freshwater drinkers, 1.16 (95% CI: 0.86, 1.56) times among the moderate‐saline water drinkers, and 1.93 (95% CI: 1.35, 2.76) times higher among the severe‐saline water drinkers in the fully adjusted model (Table 3).

**Table 3:** Odds Ratios of Neonatal and Infant Mortality Among Different Drinking Water Salinity Drinkers Relative to the Mild‐Salinity Water Drinkers

Water salinity categories	Model 1^a^	Model 2^b^	Model 3^c^
	*β* (95% CI)	*β* (95% CI)	*β* (95% CI)
Neonatal mortality			
Mild‐saline water	referent	referent	referent
Freshwater	1.55 (1.21, 1.98)	1.55 (1.21, 1.98)	1.37 (1.01, 1.84)
Moderate‐salinity water	0.94 (0.69, 1.28)	0.95 (0.70, 1.30)	1.04 (0.75, 1.45)
Severe‐salinity water	1.58 (1.07, 2.35)	1.60 (1.08, 2.38)	1.77 (1.17, 2.68)
Infant mortality			
Mild‐saline water	referent	referent	referent
Freshwater	1.61 (1.29, 2.02)	1.60 (1.28, 2.01)	1.43 (1.08, 1.89)
Moderate‐salinity water	1.09 (0.83, 1.43)	1.09 (0.83, 1.43)	1.16 (0.86, 1.56)
Severe‐salinity water	1.82 (1.30, 2.55)	1.82 (1.30, 2.56)	1.93 (1.35, 2.76)

*Note*. FAO salinity categories: Freshwater (EC: <0.7 mS/cm); Mild salinity water (EC: ≥0.7 — < 2 mS/cm); Moderate salinity water (EC: ≥2.0 — <10 mS/cm); and Severe salinity water (EC: ≥10 mS/cm). *β*, odds ratio; CI, confidence interval; EC, electrical conductivity.

a Model 1: Unadjusted

b Model 2: Adjusted for maternal age, birth order and sex of the child

c Model 3: Additionally adjusted for both parents years of education, rural or urban residence, household wealth score, improved sanitation, child's birth year, maternal marital status, depth of tubewell, geographical division, and river basin for the BDHS cluster. All co‐variates used as fixed effects

### 3.4 Drinking Water Dissolved Elements and Neonatal and Infant Mortality

**Figure 3:**
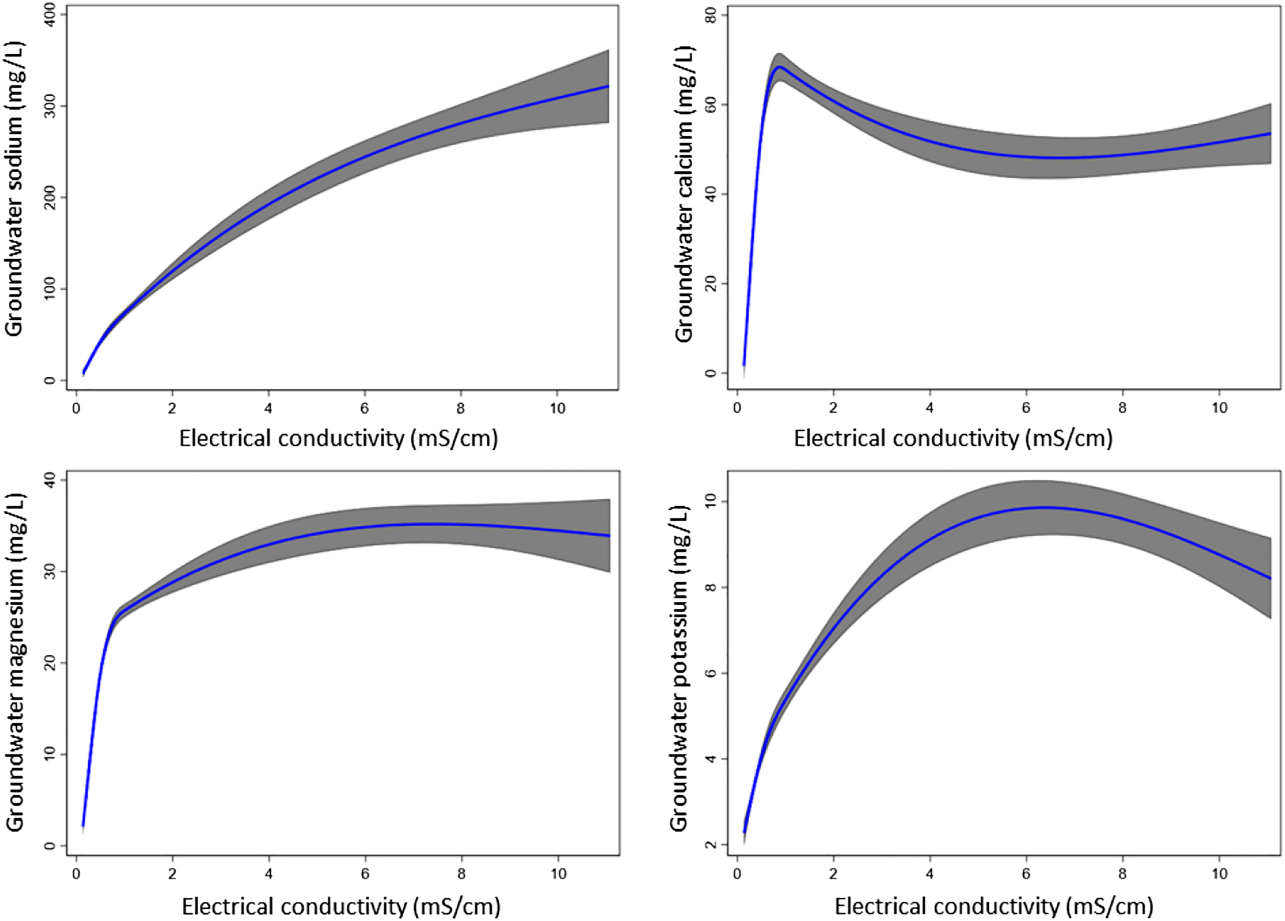
Restricted cubic spline plots (solid red line) and 95% confidence interval for the associations among groundwater electrical conductivity and specific dissolved elements in groundwater.

We found a near linear positive relationship between groundwater EC and sodium concentration (Figure 3). Groundwater calcium and magnesium concentration peaked when EC was around 1 mS/cm. However, it did not change much with a further increase in EC (Figure 3). We found an inverse U‐shaped association between groundwater EC and potassium concentration. The median groundwater sodium concentration was 25.6 (IQR: 16.1, 39.5) mg/L in freshwater, 63.4 (IQR: 37.1, 120.1) mg/L in mild‐saline water, 172.9 (IQR: 111.3, 236.2) in moderate‐saline water, and 248 (IQR: 160.0, 422.5) mg/L in severe‐saline water (Table 4). Mild‐saline water had the highest median (54.1 [1QR: 24.2, 91.5]) calcium concentration (Table 4). Median magnesium concentration was almost similar in mild salinity, moderate salinity, and severe salinity (Table 4). Restricted cubic plots suggest that increased drinking water sodium concentration was associated with increased risks for neonatal and infant deaths (Figure S2), whereas drinking water calcium was associated with lower risks for neonatal and infant deaths (Figure S3). We found declining trajectories of neonatal and infant mortality in spline plots up to 35‐mg/L water magnesium (Figure S4).

**Table 4:** Dissolved Elements Across Different Salinity Categories

Minerals	Freshwater	Mild‐salinity water	Moderate‐salinity water	Severe‐salinity water
Water sodium in mg/L, median (IQR)	25.6 (16.1, 39.5)	63.4 (37.1, 120.1)	172.9 (111.3, 236.2)	248 (160.0, 422.5)
Water calcium in mg/L, median (IQR)	27.1 (13.4, 45.6)	54.1 (24.2, 91.5)	36.9 (21.2, 66.3)	33.5 (12.3, 55.9)
Water magnesium in mg/L, median (IQR)	11.7 (6.7, 17.3)	27.0 (19.2, 38.2)	28.3 (19.3, 38.0)	29.5 (10.6, 47.5)
Water potassium in mg/L, median (IQR)	2.7 (2.0, 3.9)	5.7 (3.4, 9.3)	6.4 (4.7, 11.1)	7.1 (4.6, 12.2)

*Note*. IQR, interquartile range.

## 4 Discussion

Our analyses suggest that mild‐salinity drinking water had a protective role in the neonatal and infant deaths. Mothers reporting consumption of both freshwater and severe‐salinity water drinking water had higher risks for neonatal and infant mortality compared to the mild‐saline water drinking mothers. One possible explanation of these findings is that mothers had a lower intake of essential minerals (e.g., calcium and magnesium) through drinking freshwater. In contrast, mothers had excessive concentrations of sodium through drinking severe‐saline water. This also explains the U‐shaped association between drinking water salinity and neonatal and infant mortality—suggesting higher mortality in the freshwater and high‐salinity water ends. Our modeling from BGS‐DPHE data confirms that both drinking water calcium and magnesium were associated with lower risks for neonatal and infant mortality—but drinking water sodium was associated with higher mortality. Drinking moderate‐saline water was also associated with a protective effect on neonatal and infant mortality in unadjusted and partially adjusted models. However, these effects were attenuated in full‐multivariable adjustments.

The major cations influencing water salinity are sodium, potassium, calcium, and magnesium (Hem, 1985)—all are essential macrominerals for human needed in bulk daily amounts (Cook, 2013). High drinking water salinity (i.e., elevated EC) contains higher concentrations of these elements in drinking water (Alfarrah & Walraevens, 2018; Hoque & Butler, 2015; Mahlknecht et al., 2017; Park et al., 2012). However, these macrominerals do not contribute equally to EC at all levels. Our analyses suggest that calcium and magnesium peaks around 1 mS/cm EC, but their concentrations do not change afterward with increasing level of EC, which explains why there was lower neonatal or infant mortality among the mild‐salinity water drinkers. In contrast, the relationship between groundwater sodium and EC followed an almost a linear upward trend, suggesting severe‐saline water drinkers have very high sodium intake. The concentration of these macrominerals in drinking water is determined by the chemical properties of rocks and sediments through which groundwater percolates and interacts (Hudson, 2016). Groundwater in Bangladesh is predominantly CaHCO_3_ type, but in the coastal region where water is mostly saline, groundwater is primarily Na‐Ca‐Mg‐HCO_3_‐Cl type (Brammer, 1996). Sediments from different rivers have different chemical compositions; for example, the River Ganges‐derived sediments have higher calcium–magnesium carbonate contents than the sediments of Rivers Brahmaputra and Meghna (Brammer, 1996). It is plausible that women drinking water with high EC had higher intake of salubrious essential minerals that have been transferred to the fetus before birth or through breast milk during the nursing period. A study in coastal Bangladesh found drinking mild‐saline water was associated with higher urinary concentrations of calcium, magnesium, and sodium compared to freshwater drinkers (Naser et al., 2019). Magnesium in groundwater was associated with lower blood pressure of the Bangladeshi population (Naser et al., 2019). Studies suggest that bioavailability of essential dissolved elements from drinking water is very high (World Health Organization (WHO), 2009), and some populations in coastal Bangladesh can get up to 50% of their daily calcium and magnesium requirement by drinking 2 L of groundwater (Hoque & Butler, 2015). General diet in Bangladesh is low in calcium content (Bromage et al., 2016), and globally, magnesium concentration in general diet is declining (Kumssa et al., 2016). Therefore, drinking water may contribute an appreciable proportion of daily calcium and magnesium intake among Bangladeshi population (Hoque & Butler, 2015), and drinking fresh or low mineral water can contribute to a deficiency of these essential minerals which can have detrimental health effects.

Essential macrominerals influence many biological and enzymatic mechanisms in fetuses and infants that can influence neonatal and infant mortality (Abrams, 2007). During the intrauterine and first 6 months of life, fetuses and infants depend on their mother for these essential macrominerals. Studies suggest that calcium intake of the mother influences the bone, musculoskeletal, and cardiovascular system development (Abrams, 2007; Hacker et al., 2012; Kovacs & Kronenberg, 1997). Low calcium intake is associated with gestational hypertension (Hofmeyr et al., 2010), which is an important cause of perinatal mortality. Magnesium is a coenzyme and relevant to lung function of the neonates and infants (Caddell, 1996). Magnesium deficiency can cause bronchial constriction and can be associated with prenatal asphyxia (Seelig, 2012), one of the leading causes of neonatal death. Studies also suggest that low magnesium intake is associated with sudden infant death syndrome (Siren, 2017). Therefore, it is biologically plausible that high or low maternal intake of essential minerals through drinking water can influence neonatal and infant mortality. Calcium and magnesium supplementation during pregnancy was associated with lower pregnancy complications and neonatal mortality (Imdad et al., 2011; Zarean & Tarjan, 2017).

Infants and children need a relatively higher volume of water and a high concentration of minerals considering body weight (Sievers, 2005). According to the WHO, infants and children are an especially vulnerable population whose drinking‐water dissolved element contents need to be stabilized by adding calcium and magnesium, and other elements based on regional dietary composition (WHO, 2005). Use of freshwater or low mineral water for drinking, cooking infant food, and preparing formula milk can compromise the nutrient intake in the infants (Sievers, 2005; WHO, 2005).

Our analyses have several important limitations. First, we do not have the information on the exact drinking water salinity for the households surveyed under BDHS, as this parameter was not collected as part of the BDHS surveys. As a proxy variable for drinking water salinity, we used groundwater EC values from an interpolated map at the national scale in Bangladesh that includes groundwater EC data from coastal districts (Zahid et al., 2013). Second, the groundwater EC as well as concentrations of elements may have seasonal and temporal variations in groundwater (Dhar et al., 2008; Ourshalimian et al., 2019). Similarly, we adjusted for groundwater arsenic in final multivariate models, but groundwater arsenic data came from BGS‐DPHE survey conducted in 1998/1999, not concurrently when the mortality data were captured in BDHS surveys. Deeper aquifers (>30 m) likely had less temporal variability of elements and arsenic than the shallow aquifers (Dhar et al., 2008)—but with the lack of temporal water chemical data, we could not explore this. Third, interpolated EC from point data are smooth in spatial distribution because of geospatial interpolation method (IDW algorithm) applied for mapping. The interpolated EC values may be less or more than the actual EC values of the tubewell water used by the participants in the BDHS clusters. Furthermore, we assigned the same EC values for all households within a single cluster, whereas we know that small‐scale variations exist in groundwater elements within a short geographical distance in Bangladesh (Naser et al., 2018) due to high variability in surface geology and sediment types. Households in rural Bangladesh usually have separate tubewells for drinking water supply, suggesting that well water chemical concentrations are likely different across the households recorded in BDHS clusters. All of these factors relating to water chemistry are expected to contribute a degree of uncertainty and bias in the analyses. Moreover, we had limited data points (only 3%) in the severe‐salinity drinking water group, which may have resulted in a wide confidence interval of odds ratio at the upper end of salinity distribution. The severe water salinity problem exists only in the southwest coastal region of Bangladesh—both groundwater and pondwater of the area can be saline. If only stored rainwater or other low‐salinity water sources (e.g., pond sand filter) are not available, communities in southwest coastal Bangladesh drink saline water. Therefore, it was realistic that we had a few data points at the upper end of salinity.

## 5 Conclusion

Groundwater is the primary source of drinking water in rural Bangladesh, and elements in groundwater have important public health implications (Smith et al., 2000). Our findings indicate that both freshwater and severe‐salinity water are associated with higher infant and neonatal mortality. We recommend confirming or refuting our findings prior to any relevant policy decisions. We recommend a detailed analysis of specific minerals in common diet and drinking water among the Bangladeshi population and a comparison of bioavailability of minerals from diet and drinking water. Dissolved elements in drinking water may contribute an appreciable proportion of daily mineral intake if (1) the common diet is deficit of minerals or (2) bioavailability of minerals from food is reduced due to complex chemical forms and interactions with other chemicals in food. In contrast, elements exist in simple ionic forms in drinking water that may have higher bioavailability (WHO, 2009). If we consider bioavailability of elements from drinking water is higher, fortification of drinking water with minerals may be a more pragmatic way to provide mineral delivery to the mothers (Naser et al., 2017; Naser et al., 2019). We also recommend prospective epidemiological studies looking at the precise measurement of drinking water dissolved elements and mineral concentrations in mothers’ blood, urine, or other biological matrices and then linking up with perinatal complications and neonatal death. If confirmed mineral contents are associated with low neonatal and infant mortality, mineral fortification of fresh, less‐mineralized drinking water can be an important public health intervention to reduce neonatal and infant mortality in Bangladesh.

## Conflict of Interest

The authors declare no conflicts of interest relevant to this study.

## Supporting information



Supporting Information S1
