# Supplementary material for: Modeling the Relationship of Groundwater Salinity to Neonatal and Infant Mortality From the Bangladesh Demographic Health Survey 2000 to 2014
Source: Geohealth. 2020 Feb 17;4(2):e2019GH000229. doi: 10.1029/2019GH000229 (PMC7025866; doi:10.1029/2019GH000229)
Supplement: Supplementary file 1 — Supporting Information S1 [file GH2-4-e2019GH000229-s001.docx]

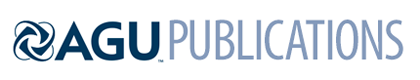


*GeoHealth*

Supporting Information for

**Modeling the relationship between groundwater salinity to neonatal and infant mortality from the Bangladesh Demographic Health Survey 2000 to 2014**

Abu Mohd Naser^1^, Qiao Wang^2^, Mohammad Shamsudduha^3,4^, Gnanaraj Chellaraj^2^, George Joseph^2^

^1^ Hubert Department of Global Health, Rollins School of Public Health, Emory University, Atlanta, Georgia, USA

^2^ World Bank, Washington DC, USA

^3^ Institute for Risk and Disaster Reduction, University College London, UK

^4^ Department of Geography, University of Sussex, Brighton, UK

**Contents of this file**

Figures S1 to S5


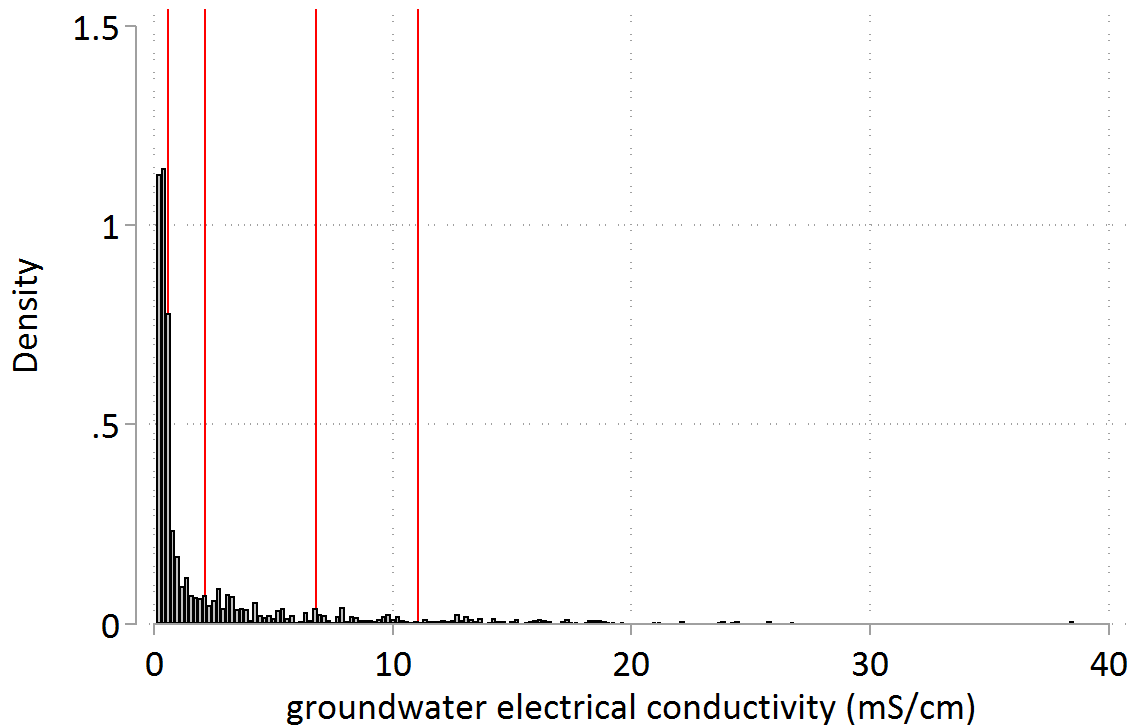
Figure S1: Histogram and density plots for groundwater electrical conductivity. Red vertical lines denote the distributions of electrical conductivity data at 50%ile (median), 75%ile, 90%ile and 95%ile.


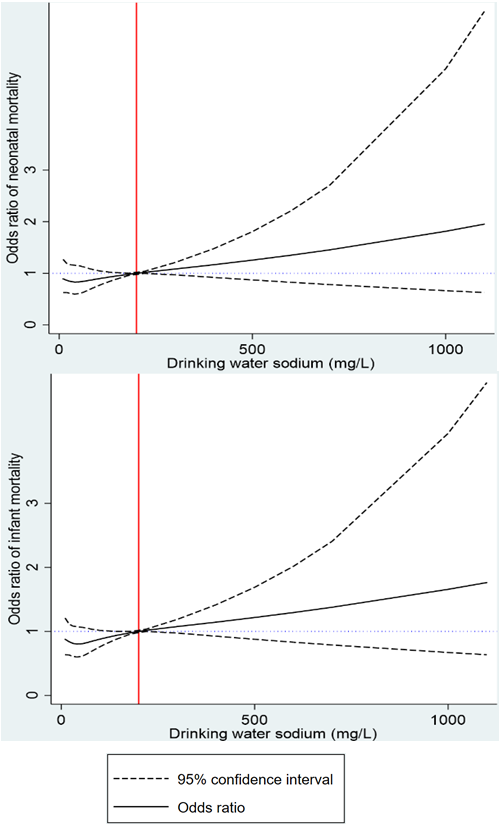


*Figure S2: Restricted cubic spline plots (solid lines) and 95% confidence interval (dashed lines) for the association between drinking water sodium concentration and neonatal and infant mortality. Restricted cubic splines were plotted at electrical conductivity cut-points of 5%ile, 35%ile, 65%ile, and 95%ile. Red solid vertical line (at sodium= 200 mg/L) indicates the Bangladesh standard for drinking water sodium against which odds ratios were calculated. Odds ratio = 1 denoted by blue dotted line.*


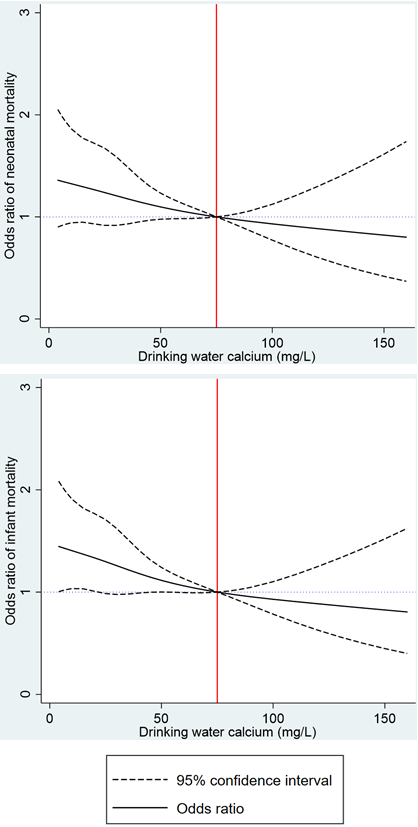


*Figure S3: Restricted cubic spline plots (solid lines) and 95% confidence interval (dashed lines) for the association between drinking water calcium concentration and neonatal and infant mortality. Restricted cubic splines were plotted at electrical conductivity cut-points of 5%ile, 35%ile, 65%ile, and 95%ile. Red solid vertical line (at calcium= 75 mg/L) indicates the Bangladesh standard for drinking water calcium against which odds ratios were calculated. Odds ratio = 1 denoted by blue dotted line.*


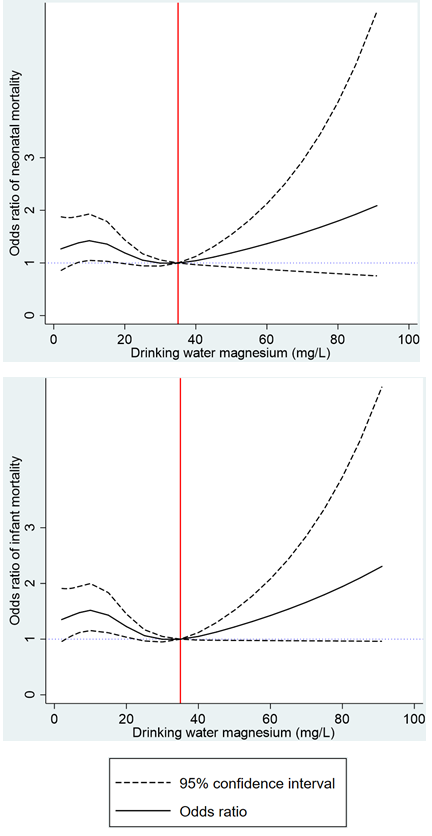


*Figure S4: Restricted cubic spline plots (solid lines) and 95% confidence interval (dashed lines) for the association between drinking water magnesium concentration and neonatal and infant mortality. Restricted cubic splines were plotted at electrical conductivity cut-points of 5%ile, 35%ile, 65%ile, and 95%ile. Red solid vertical line (at magnesium= 35 mg/L) indicates the Bangladesh standard for drinking water magnesium against which odds ratios were calculated. Odds ratio = 1 denoted by blue dotted line.*


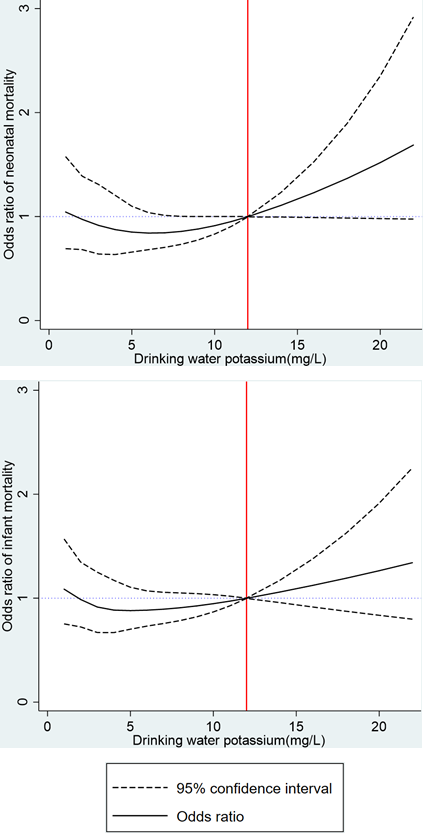


*Figure S5: Restricted cubic spline plots (solid lines) and 95% confidence interval (dashed lines) for the association between drinking water potassium concentration and neonatal and infant mortality. Restricted cubic splines were plotted at electrical conductivity cut-points of 5%ile, 35%ile, 65%ile, and 95%ile. Red solid vertical line (at potassium= 12 mg/L) indicates the Bangladesh standard for drinking water potassium against which odds ratios were calculated. Odds ratio = 1 denoted by blue dotted line.*
